# Exploring the implementation of an outreach specialist program for nursing home residents in Macao: A multisite, qualitative study

**DOI:** 10.3389/fpubh.2022.950704

**Published:** 2022-09-29

**Authors:** Zhifeng Cen, Junlei Li, Hao Hu, Ka Cheng Lei, Cheng I Loi, Zuanji Liang, Tek Fai Chan, Carolina Oi Lam Ung

**Affiliations:** ^1^State Key Laboratory of Quality Research in Chinese Medicine, Institute of Chinese Medical Sciences, University of Macau, Taipa, Macao SAR, China; ^2^Department of Public Health and Medicinal Administration, Faculty of Health Sciences, University of Macau, Taipa, Macao SAR, China; ^3^Macao Society for Medicinal Administration, Taipa, Macao SAR, China

**Keywords:** nursing home, public-private partnership, Macao, outreach specialist program, inter-disciplinary, elderly, patient-centered care, cost-effectiveness

## Abstract

**Background:**

The “Specialist Medical Outreach Project (SMOP)” involving inter-disciplinary hospital-based healthcare professionals is a government initiative that aims to provide integrative specialist care to high-risk residents at the nursing homes. However, research exploring the implementation and impact of SMOP is lacking. This study aimed to evidence the impact of SMOP on the quality of care at the nursing home and the key contextual determinants influencing SMOP outcomes.

**Method:**

Semi-structured key informant audio-recorded face-to-face interviews were conducted with eight managers, six doctors, 28 nursing staff, and seven pharmacy staff at the nursing homes participating in the SMOP to collect insights about how SMOP was operated and performed, and the impact of SMOP as observed and expected. Participants were recruited with purposive sampling. A thematic analysis approach was employed and key themes were identified using open coding, grouping, and categorizing.

**Results:**

Forty-nine interviews were conducted. Thematic analysis identified three principal themes: the overall perception about SMOP, the benefits as observed; and the areas of improvement. Together with the 10 subthemes, the results highlighted the expectations for SMOP to address the unmet needs and promote patient-centered care, and the benefits of SMOP in supporting effective use of resources for the nursing home, reducing the risks of adverse events for the residents, promoting communication and capacity building for the healthcare providers and facilitating efficient use of healthcare resources for the health system. Requests for more frequent visits by a larger inter-disciplinary specialist team were raised. Careful staff and workflow planning, and mechanisms for data-sharing and communication across care settings were deemed the most important actions for improvement.

**Conclusion:**

It is a general perception that the SMOP is beneficial in enhancing the quality of care for high-risk residents in the nursing home in Macao. Cross-sector inter-disciplinary collaboration and efficient data-sharing and communication mechanism play a crucial role in ensuring the success of the program. A robust assessment framework to monitor and evaluate the cost-effectiveness of the program is yet to be developed.

## Introduction

Nursing homes play an increasingly important role in offering high level of medical care to their residents especially to those who have complicated and serious health concerns (1). However, multifaceted complications challenging the provision of medical care exist at the interface between the residents and the setting of nursing homes. On one hand, the elderly residents are often frail, multi-morbid population featured with compromised physical and cognitive dysfunction that required complicated medical and pharmacological treatment (2, 3). On the other hand, the challenges of caring for the residents at the nursing homes are further compounded by at least 3 major institute-based factors: constrained funding, inadequate staffing and soaring workloads (4). Nursing home residents were subject to high risks of costly hospitalization and prolonged hospital stays (5).

How to optimize the quality of medical care in nursing homes and effectively minimize the residents' risks of avoidable emergency department visits and hospitalization has driven the development of different healthcare-related outreach programs initiatives (6). Such outreach programs referred to “*a temporary and mobile project that engages the community to collaborate in undertaking the purposeful health intervention to reach the population at health risk*” (7). Outreach teams usually comprised of inter-disciplinary healthcare providers and the interventions usually involved comprehensive health assessments and direct care of the residents (6, 8–12). Inter-disciplinary meetings between the outreach team and the nursing home staff often focused on problem-solving and case management for residents (6, 11, 12). Education and training may also be provided by the outreach team to the nursing home staffs (6, 11–13).

Outreach programs for the nursing homes were usually designed to encourage institutional cooperation across different care settings and facilitating the medical accessibility by individuals at risks (7). Studies have found that such outreach programs involving geriatricians resulted in fewer hospitalizations among the residents served (10, 14). Another study also found that pharmacist-led medication review provided through an outreach program for nursing home residents helped reduce hospital admissions and total estimated drug cost (15). Benefits in coordinating care and capacity building among the residential aged care staff (16), reducing the burden of elders' complex symptoms of mental illnesses, improving their function and quality of life and alleviating the problems of medical staff shortage in the nursing home (17) have also been reported. Although the evidence of improved outcome for the residents due to various outreach programs continues to emerge, little has been reported about the development and implementation of process (6, 18).

In Macao, a similar outreach program called the “Specialist Medical Outreach Project (SMOP)” has been initiated by the government to serve the residents staying at the subsidized nursing home since 2018 (19). This was one of the actions taken under the “2016-2025 Ten-Year Action Plan for Elderly Services”, which aimed to ensure adequate protection and appropriate support for the elderly in terms of physical needs, as well as physical and mental safety. Aging population remains a key challenge to the sustainable development of the city as the proportion of people aged 65 or above is expected to exceed 20.0% in 2031 (20). This elderly population group enjoys free healthcare (medical services and medicines) and are entitled to stay at the government-subsidized nursing homes for free or at very low costs when needed.

In the SMOP, specialists of geriatric medicine, internal medicine, psychiatry, and emergency medicine, as well as pharmacists and physiotherapists from the public hospital paid regular visits to the nursing homes to consult the residents and conduct inter-disciplinary consultation meetings with the nursing home frontline staff (19). This reinforced patient-centered care and represented an opportunity for healthcare providers from different sectors to engage in formal discussions over diagnostic and therapeutic strategies and decide on medical decisions collectively for nursing home residents. Residents who frequently needed to travel to the public hospital to consult the same specialists were prioritized to receive the SMOP services. The purpose of SMOP was to optimize the convenience of accessibility to integrative care, ensure early detection of signs of deterioration of health conditions for timely management, and thus minimize avoidable hospitalization. At a higher-level perspective, since the government is in charge of supervising and funding both the subsidized nursing homes and the provision of healthcare services to elderly population, the SMOP was also set to improve the cost-effectiveness of government resources through collaborative effort between the health and social welfare sectors (19).

To be able to use SMOP as a tool effectively to achieve the expected benefits at the individual, nursing home and government levels, it is important to have a good understanding about how it has been implemented, the current performance, and areas of improvement. This is in alignment with previous recommendations that for complex interventions whereby the impact is heavily dependent on the interaction between the intervention and its context, it is important to examine the implementation process, the way an interventions worked, the mechanisms of impact and the contextual factors (21) in order to contribute to the broader development, transfer, implementation and scaling-up of the intervention (22). In addition, despite years of operation through which 4,593 SMOP consultations had been provided and 16,078 person-time served (23), the SMOP experiences remained under-reported. As such, this study aimed to narrate the experiences of developing and implementing the SMOP in Macao. It is anticipated that the study findings will not only help to shed light on how SMOP was experienced by the nursing home staff, but also complement the current understanding of how health-related outreach programs perform for nursing homes in general.

## Materials and methods

This study employed semi-structured interviews which were recommended for acquiring rich information about the experiences of receiving SMOP services at the nursing home (24). The study protocol was approved by the Research Ethics panel of the University of Macau (reference number: SSHRE21-APP001-ICMS). The study results are reported in compliance with the Consolidated Criteria for Reporting Qualitative research (COREQ) in the following (25).

### Recruitment

Purposive sampling strategy was employed to recruit participants. Invitation, in which the Participant Information Statement and the Interview guide written in both English and Chinese were attached, was sent to all 11 nursing homes participating in SMOP using their contact information obtained from the official website of the Macao Social Welfare Bureau. These nursing homes spread out widely across different parts of Macao caring for similar elderly population groups.

The persons-in-charge of the nursing homes, who were often in the position to approve their staff to take part in studies which might involve the disclosure of any work-related information and responsible for making staff and logistic arrangement for SMOP services, were invited to nominate at least three staff members to take part in a 30-min interview separately. They were also asked to share the Participant Information Statement and the Interview guide with the eligible staff members.

Staff members who were charged with management duties, healthcare professionals or frontline staff having direct experiences with SMOP and could communicate in either English or Chinese were deemed eligible for this study. A minimum of three staff members with preferably different professional backgrounds from each nursing home (one manager, one doctor, and one nursing staff) were anticipated to provide a comprehensive viewpoint about SMOP without causing too much disruption to the daily operation of the nursing homes. Nursing homes equipped with pharmacy staff were encouraged to also nominate pharmacists or pharmacy technicians to participate. No exclusion criteria were defined prior as potential participants were recruited strategically to obtain insight from a range of essential stakeholder perspectives. By recruiting participants with diverse backgrounds as many as possible, the triangulation process was to render a holistic picture of the receiving side about SMOP and to prevent improper reliance on selective data sources.

### Data collection

For the purpose of this study, individual interviews were preferred over other qualitative research methods (such as focus groups) mainly due to: (1) the advantage of detailed exploration of the participants experiences with SMOP; (2) the difficulties of engaging a number of staff members at the same time for focus group during their work hours; (3) the possible differences in the time each participant would be able and willing to spend on participating in this study; and (4) the flexibility of interview scheduling according to the participants' availability.

Interviews were conducted with all nominees who expressed an interest in participating and gave their informed written consent in English or Chinese. Before the start of each interview, the study background and the consent form was explained to the participants. Participants were free to decide whether they would like to participate. Only when they had signed the written consent form, written in English or Chinese, would the interview begin. All the semi-structured interviews were conducted face-to-face in the private conference room at the nursing home where the participants worked.

Participants were asked to share their options about the overall design of SMOP, the expected and observed outcome, the key factors influencing the program implementation and outcome, and the potentials for the SMOP to sustain and expand. Open-ended questions were developed in consultation with related literature about the role of coordinated care, the potential benefits, and the challenges and facilitators of outreach program alike (8, 26–29) to understand the processes and the potential causes of observations (30). The wordings used in each question were as generic as possible to allow easy understanding of the aspects of SMOP which were of interests to this study regardless of the participants' professional background.

A team of six investigators (including five master students and one PhD student in medicinal administration or pharmacy) who were highly-trained in qualitative studies were responsible for conducting the interviews. All investigators were fluent in English and Chinese, and had experiences in conducting qualitative research. An interview protocol including the interview questions was designed for the investigators to comply with during each interview (Supplementary File 1). Prior to the start of the interviews, all investigators received a group briefing by the senior author (COLU) about the purpose of this study and the Interview Protocol. During the briefing, role-plays were also performed by the investigators and assessed by the senior author to ensure the mutual understanding of the procedure. Each interview was closely monitored by the senior author upon completion to address any concerns the investigators might have and to identify any possible issues with conducting the interviews for timely interventions. The investigators were equally divided into 2 groups. Each interview was conducted by either one of the investigator groups, during which one investigator led the conversation with the participant while the other two investigators were responsible for recording field notes, key discussion points and non-verbal expression of the participant. It was anticipated that the above actions would collectively help to optimize the reliability and credibility of the interviews.

A pilot testing with two pharmacists and one nursing staff who had a professional background in nursing home was conducted by the investigators separately. The pilot study was conducted to help refine and target the interview questions to be more specific in preparation for the stakeholder interviews. At the same time, the pilot study also served as an opportunity for the investigators to develop their communication skills around the questions to be asked, to collect more background information about SMOP, to speculate how to conduct the interviews more effectively, and to verify the approach of data analysis (31–34). Based on the feedback from both interviewees and participants collected during the pilot testing, minor revisions were made to the wordings of the interview questions to finalize the complete interview guide.

The interviews were conducted either in Chinese or English according to the participants' preference and in accordance to the Interview Protocol. Each interview was audio-recorded and conducted until saturation was reached for the key emergent themes (30). No participants were excluded due to language barriers as nursing home staff were fluent in at least one of the two languages. Some participants were known to the investigators through pharmacists' professional networks but otherwise not related and none of the investigators had any personal relationship with the participants. Each interview lasted for 20–50 min (an average of 35 min). All interviews were conducted between February and March in 2021.

### Data analysis

The audio recordings of the interviews were transcribed verbatim in the language used in the interviews before analysis with an Excel file which was developed for the codes of the transcripts to be tabulated and organized. During transcription, two investigators separately listened to all the interviews and read the transcripts and observation notes multiple times, recorded key and recurrent ideas as they emerged, and generated initial codes to capture the meaningful fundamental element of the data. The coding results of each transcript was input into the Excel file, compared and negotiated among the pair. The analysis of data was conducted following the Data Analysis Protocol designed for this study (Supplementary File 2).

Thematic analysis approach was adopted in the attempt to gain a better understanding about the linkage between the needs and program planning that contributes to the efficiency and improves outcomes (35). All the coding results were pooled, and all the codes were categorized into themes and sub-themes by the six investigators together. To help ensure credibility, dependability and confirmability of the findings, member check of the coding results of individual transcripts and the pooled dataset was offered to the participants (36). Disagreements on the coding results were subject to final confirmation by the senior author. The information obtained from the interviews was de-identified and the participants were only referred with his/her role in the coded nursing home in the manuscript. Prior to inclusion for reporting, all the quotes originally written in Chinese were translated into English by three investigators and back translated by the other three investigators to verify the accuracy of the translation. The final version of the translation was confirmed by the senior author.

## Results

### Participant characteristics

Eight of the 11 invited nursing homes participated in this study. They were located in various parts of Macao including residential areas, suburban and remote areas. The three nursing homes which did not accept the invitation explained that they were too busy to participate due to constant shortage of staff. A total of 49 semi-structured interviews were conducted with eight managers, six doctors, 28 nursing staff (16 nurses or 12 nurse assistants), and seven pharmacy staff (two pharmacists and five pharmacy technicians) (Table 1). All the staff members nominated to participate took part in the study and there was no withdrawal.

**Table 1:** Participant characteristics (*n* = 49).

	**ID**	**Nursing home**	**Role**	**Gender**	**Number of years working in the nursing home**
1	Nursing home 1_01	Nursing home 1	Doctor	Female	5 years
2	Nursing home 1_02	Nursing home 1	Pharmacist	Female	3 years
3	Nursing home 1_03	Nursing home 1	Pharmacy technician	Female	3 years
4	Nursing home 1_04	Nursing home 1	Manager	Female	5.5 years
5	Nursing home 1_05	Nursing home 1	Nurse	Female	5 years
6	Nursing home 2_01	Nursing home 2	Doctor	Male	2 years
7	Nursing home 2_02	Nursing home 2	Nurse	Female	2.5 years
8	Nursing home 3_01	Nursing home 3	Doctor	Male	1.5 years
9	Nursing home 3_02	Nursing home 3	Pharmacy technician	Female	1 year
10	Nursing home 3_03	Nursing home 3	Nurse	Female	1 year
11	Nursing home 3_04	Nursing home 3	Manager	Female	9 years
12	Nursing home 4_01	Nursing home 4	Manager	Female	7 years
13	Nursing home 4_02	Nursing home 4	Manager	Female	5 years
14	Nursing home 5_01	Nursing home 5	Doctor	Male	5 years
15	Nursing home 5_02	Nursing home 5	Pharmacy technician	Male	6 years
16	Nursing home 5_03	Nursing home 5	Nurse	Female	2 years
17	Nursing home 5_04	Nursing home 5	Nurse	Female	3.5 years
18	Nursing home 5_05	Nursing home 5	Nurse	Female	<1 year
19	Nursing home 5_06	Nursing home 5	Nurse	Female	<1 year
20	Nursing home 5_07	Nursing home 5	Manager	Female	11 years
21	Nursing home 5_08	Nursing home 5	Manager	Female	7 years
22	Nursing home 5_09	Nursing home 5	Manager	Male	6 years
23	Nursing home 6_01	Nursing home 6	Nurse	Female	4 years
24	Nursing home 6_02	Nursing home 6	Nurse	Female	<1 year
25	Nursing home 6_03	Nursing home 6	Nurse	Female	<1 year
26	Nursing home 6_04	Nursing home 6	Nurse assistant	Female	6 years
27	Nursing home 6_05	Nursing home 6	Nurse assistant	Female	6 years
28	Nursing home 6_06	Nursing home 6	Nurse assistant	Female	15 years
29	Nursing home 6_07	Nursing home 6	Pharmacy technician	Female	1 year
30	Nursing home 7_01	Nursing home 7	Nurse	Male	1.5 years
31	Nursing home 7_02	Nursing home 7	Nurse	Male	2 years
32	Nursing home 7_03	Nursing home 7	Manager	Female	6 years
33	Nursing home 7_04	Nursing home 7	Nurse	Male	1.5 years
34	Nursing home 7_05	Nursing home 7	Nurse	Male	2 years
35	Nursing home 7_06	Nursing home 7	Nurse	Female	3 years
36	Nursing home 7_07	Nursing home 7	Nurse assistant	Female	3 years
37	Nursing home 7_08	Nursing home 7	Nurse assistant	Female	3.5 years
38	Nursing home 7_09	Nursing home 7	Nurse assistant	Female	7 years
39	Nursing home 7_10	Nursing home 7	Nurse assistant	Male	14 years
40	Nursing home 7_11	Nursing home 7	Nurse assistant	Male	20 years
41	Nursing home 7_12	Nursing home 7	Nurse assistant	Female	<1 year
42	Nursing home 7_13	Nursing home 7	Nurse assistant	Male	<1 year
43	Nursing home 7_14	Nursing home 7	Nurse assistant	Female	<1 year
44	Nursing home 7_15	Nursing home 7	Nurse assistant	Female	<1 year
45	Nursing home 7_16	Nursing home 7	Pharmacist	Male	1.5 years
46	Nursing home 7_17	Nursing home 7	Doctor	Female	<1 year
47	Nursing home 8_01	Nursing home 8	Doctor	Female	4 years
48	Nursing home 8_02	Nursing home 8	Nurse	Female	1.5 years
49	Nursing home 8_03	Nursing home 8	Pharmacy technician	Female	3.5 years

### Themes

According to the thematic analysis, three principal themes had been identified: the overall perception about the SMOP, the benefits of the SMOP as observed; and the areas of the improvement of SMOP. Together with the 10 subthemes and 54 codes, the results collectively reflected the totality of the participants' experiences with SMOP as shown in Table 2 (For examples of supporting quotes, please refer to the Supplementary File 3).

**Table 2:** Thematic analysis of the qualitative data.

**Themes**	**Subthemes**	**Codes**
Theme 1: Overall perception about the SMOP	Subtheme 1.1: Addressing the needs	1. *Lack of expertise* 2. *Lack of manpower* 3. *Time constraints* 4. *Difficulties in logistics arrangement* 5. *Transportation challenges* 6. *Waiting time*
	Subtheme 1.2: Sharing information among caregivers	1. *Blood test results* 2. *New diagnosis* 3. *Changes in treatment* 4. *Adverse drug reactions* 5. *New signs and symptoms* 6. *New health concerns for the residents*
	Subtheme 1.3: Access to the experts	1. *Visits by specialists* 2. *Access to specialists' advice* 3. *Cross-sector collaboration* 4. *Learning opportunity*
Theme 2: Benefits of the SMOP as experienced or expected	Subtheme 2.1: At the nursing home level	1. *Quality of care* 2. *Safety* 3. *Effective use of resources* 4. *Accuracy* 5. *Patient-centered care* 6. *Effective use of resources*
	Subtheme 2.2: At the resident level	1. *Early detection of critical health issues and timely referral* 2. *Reduced unplanned hospital admission* 3. *Improved disease control* 4. *Improved clinical outcome* 5. *Reduced risks of adverse events* 6. *Reduced visits to ER* 7. *Reduced hospitalization*
	Subtheme 2.3: At the professional level	1. *Communication* 2. *Collaboration* 3. *Inter-disciplinary healthcare team* 4. *Capacity building for nursing home staff* 5. *Timely update of residents' conditions* 6. *Reduced error in transferring residents' clinical data*
	Subtheme 2.4: At the health system level	1. *Integration of healthcare resources* 2. *Prioritization of healthcare resources* 3. *Improvements in elderly care* 4. *Reduction of avoidable burden on healthcare system* 5. *Prevention of drug wastage* 6. *Capacity building for nursing home staff and SMOP team members*
Theme 3: Areas of improvement of SMOP	Subtheme 3.1: The design of the SMOP	1. *Frequency of visits* 2. *Involvement of more specialists in the SMOP team* 3. *Greater support for psychiatric and mental health of the residents* 4. *Case discussion*
	Subtheme 3.2: Preparation of the nursing home	1. *Better workflow* 2. *Staffing arrangement prior to and during SMOP team visits* 3. *Preparation of residents' health records* 4. *Screening criteria for residents receiving SMOP services*
	Subtheme 3.3: A continuous communication and data sharing mechanism	1. *Feedback loop* 2. *Cross sector communication* 3. *Data sharing mechanisms* 4. *Access to electronic medical record* 5. *Means of cross-sector communications*

*Theme 1: Positive perception about the SMOP*

When asked about their general perception of the SMOP, participants shared their thoughts mainly from three perspectives: their needs, their anticipations and the importance of an inter-disciplinary team. All the participants were positive about the SMOP and indicated a high level of recognition for the government input through the SMOP.

*Subtheme 1.1: Addressing the needs*

Participants indicated that they had to look after residents who required different levels of daily and medical care. Residents who had multi-comorbidities would need to see different specialists on a regular basis. Arranging frail elderly to travel to and from the hospital was highly resource-consuming. More importantly, it could be difficult for the residents to endure the long travel and waiting time. Knowing that the government would send a team of specialists to reach out and check up on their residents, all participants had high regards for the SMOP.

“*We are so pleased to have the SMOP now and to see how the SMOP has helped us and our residents where we need the help the most.” (Nursing home 5_07, manager)*

“*The integrated expertise support is exactly what we need to care for our very sick residents.” (Nursing home 4_01, manager)*

*Subtheme 1.2: Sharing information among caregivers*

Many participants, especially the healthcare professionals, anticipated that the SMOP would help foster communication among them and promote collaboration across different care settings.

“*Now we (the doctor and the specialists) can actually discuss about the cases and decide on the treatment together whenever the SMOP team is here.” (Nursing home 5_01, doctor)*

“*The opportunity is for us to share with the pharmacists in the SMOP team any adverse drug events we observe especially when new medicines are prescribed by the specialists. Having a pharmacist in the SMOP team means that we can now work closer together to ensure drug safety.” (Nursing home 7_16, pharmacist)*

*Subtheme 1.3: Access to the experts*

Participants believed that the inter-disciplinary team allowed the SMOP to bear at least two primary functions: assessing the residents' conditions and making referral whenever needed, and conducting discussion with and training to the healthcare staff in the nursing home. Accordingly, the SMOP team purposively included specialists most needed by geriatric patients such as specialists of geriatric, internal medicine, psychiatry, and emergency department, in addition to nurses and clinical pharmacists.

“*Discussion with different specialists at the same time is also a very good leaning opportunity for us.” (Nursing home 1_01, doctor)*

“*Pharmacy staff can also learn from them (the clinical pharmacist in the SMOP team) how to evaluate and optimize the drug regimens.” (Nursing home 5_02, pharmacy technician)*

*Theme 2: Benefits of SMOP as observed*

Participants reported a range of benefits observed at their workplace as a results of having regular visits by an inter-disciplinary specialist team through the SMOP.

*Subtheme 2.1: At the nursing home level*

The SMOP arrangement could help reduce the manpower and resources needed for mobilizing residents across different healthcare settings. Such resources could be used to cover other areas of concerns and improve the overall efficiency of the nursing home. Most nursing homes had full time in-house doctors and smaller nursing homes had visiting doctors who visited the nursing home several times a week. The SMOP arrangement was especially important for nursing homes which had only one in-house doctor or even just visiting doctor.

“*More importantly, the SMOP saved us a lot of resources and manpower to transport our residents to the hospital and back. Such resources can be diverted to cover other areas of concerns.” (Nursing home 6_06, nurse assistant)*

In addition, having the visits by the SMOP team also prompted closer attention to any potential issues with the medical care provided to the residents and helped enhance the quality of medical care. As explained by the medical and nursing staff, the SMOP was considered an additional driving force to ensure the quality of record-keeping, to screen patients for risks of uneventful incidents, and to properly prepare health records of high-risks patients who would be consulted during the SMOP. This represented not only the needs to cross check residents' conditions and records as part of the preparation work for the SMOP visits, but also the opportunities for developing a more rigorous practice among the nursing home staff.

“*So you see, the SMOP has actually prompted us to pay closer attention to the most needy residents before, during and after the SMOP team visit. In a way, the SMOP helps us double or even triple check our services.” (Nursing home 1_04, manager)*

*Subtheme 2.2: At the resident level*

Participants mentioned that, with the support of the SMOP team, critical health issues in some residents had been detected early enough for timely referral to be made to avoid deterioration of the health conditions and emergence of disabilities due to untimely treatment or failure to detect the disease in time. At times, the SMOP team would also pay *ad hoc* visits when the conditions of the residents had become critical. The intervention provided by the SMOP team, as speculated by the participants, could help prevent avoidable emergency room visits for the residents.

“*In addition to the planned residents to be visited, the team also will visit other residents who indeed need a visit. If there were an urgent situation, we would discuss with the SMOP team to try to resolve the problem sooner.” (Nursing home 7_03, manager)*

“*But more often, potential issues can be picked up much sooner through discussion for actions to be taken in a timely manner to avoid deterioration of the health condition and the residents did not have to end up in the emergency room.” (Nursing home 6_05, nurse assistant)*

*Subtheme 2.3: At the professional level*

Participants believed that the visits by the SMOP team could effectively facilitate cross-sector communication and collaboration which was beneficial to the provision of quality care. Contacting specialists to discuss about the residents' health conditions had been a challenge due to a lack of an effective communication mechanism prior to SMOP.

“*The SMOP is an opportunity for us to get access to the residents' latest clinical readings especially after recent discharge so that we can look after our residents more appropriately.” (Nursing home 2_02, nurse)*

“*I believe our input is also useful for the specialists' decision-making. It feels like we have a partner to communicate and work with.” (Nursing home 8_01, doctor)*

Having professional dialogues with the SMOP team also encouraged healthcare providers in the nursing home to be more proactive in developing their competence and provide professional services, and to play a role in the inter-disciplinary healthcare team to achieve patient-centered care. The SMOP also took the opportunity to improve their expertise as suggested by some of the doctors, nurses and pharmacists.

“*Pharmacists of the SMOP team will come here and give us feedback. The SMOP specialists also provide feedback to our doctors.” (Nursing home 1_02, pharmacist)*

“*I can see that the SMOP continues to evolve to better match our needs.” (Nursing home 5_01, doctor)*

*Subtheme 2.4: At the health system level*

Participants believed that the SMOP could help to refocus the resources to more needy residents and to reduce unnecessary visits to the emergency department and hospitalization. Therefore, the efficient use of the overall healthcare resources could be promoted resulting in refocus to more needy residents.

“*Every time they visit, they will prioritize their time to the residents who have been previously screened as high risk. Whenever they have time, they will also check on residents who are more seriously ill, or bed-ridden. In a way, they (the SMOP team) also take the chance to help reduce the use of health resources in the hospital setting. I can see that the SMOP continues to evolve to better match our needs.” (Nursing home 5_01, doctor)*

As explained by multiple participants, medication review conducted by the SMOP team at the nursing home could help prevent drug wastage and optimize medication regimens. Without the SMOP, the residents needed to pay separate visits to different specialists to get the refills of their prescription medicines, posing high risks of polypharmacy. Also, the health conditions of the residents might vary when they were back in the nursing home. Changes to the treatment regimens might be ordered by the doctors at the nursing home whenever deemed necessary. However, the specialists might not be informed about the changes and continued to prescribe the same medications in the residents' next visits.

“*Working with the SMOP team means that we are at a better position to help optimize the treatment regimen, ensure the proper use of medicine and minimize any drug wastage for our residents.” (Nursing home 4_01, manager)*

*Theme 3: Suggestions to improve the outcome of the SMOP*

Participants elaborated on the actions needed to optimize the outcome of the SMOP mainly from three perspectives: the design of the SMOP, the preparation of the nursing home, and the need for a data sharing and continuous communication mechanism.

*Subtheme 3.1: The design of the SMOP*

Although the visit of the SMOP team had indeed induced increased workload for the staff in the nursing home, it was their general belief that the SMOP was beneficial in improving the quality of care for the residents. As such, most participants were hoping to see more frequent visit by the SMOP team and suggested the inclusion of more specialists in the SMOP team to address the unmet needs of some resident subgroups such as those with mental health disorders.

“*I hope the SMOP team can visit us more often and we can get more support for residents with mental illnesses.” (Nursing home 3_03, nurse)*

“*Of course, to the residents, having more specialists in the SMOP team is of course better.” (Nursing home 8_03, pharmacy technician)*

*Subtheme 3.2: Preparation of the nursing home*

Participants realized their role in coordinating the SMOP operation and supporting the SMOP team was to get the residents' profile that showed a clear medical and medication history well-prepared and participate in the consultations and discussion during the visit. However, the current workflow and staffing might have some negative impact warranting adjustment.

“*The preparation work of the SMOP team visit is a time-consuming task and adds on the work pressure that we already have to endure. Better workflow or even IT tools are needed to help alleviate our workload.” (Nursing home 8_02, nurse)*

“*I am trying to adjust the staffing so that our frontline staff are able to work with and benefit from the SMOP team whenever they visit.” (Nursing home 4_01, manager)*

*Subtheme 3.3: A continuous communication and data sharing mechanism*

Participants emphasized on the importance of an effective communication mechanisms so that the residents' most updated information could be shared and accessed by practitioners from different settings and the healthcare providers could engage in discussion whenever deemed necessary for the care of the residents.

“*I think, with the residents' consent, having a proper data sharing mechanism between the nursing home and the hospital is important so that we can share, update and get access to the residents' key clinical notes and blood test results.” (Nursing home 2_02, nurse)*

“*Given the long waiting list to specialty consultation or surgery, if there is already a communication mechanism so that alert can be raised in case of raised urgency, the management of the residents can be much better informed and, whenever appropriate, prioritized. (Nursing home 5_08, manager)*

## Discussion

The results of the analysis of the 49 interviews with managers and frontline staff of the nursing homes conducted in this study identified a generally positive attitude toward the government-initiated SMOP and a range of opinions about how SMOP could help promote patient-centered care for the residents. These results are consistent with previous experiences of outreach interventions at the nursing homes that benefited coordinated care, access to skilled care providers, capability building, risk stratification, partnerships and comprehensive geriatric assessment (37). The results also provided insight into nursing home staff's anticipation for continuous development of SMOP and perceived importance of data-sharing and communication across care settings. However, key factors such as continuous government commitment and an evaluation mechanism of the program outcome were rarely mentioned.

This study reaffirmed the common phenomena that the population of residents of nursing homes were often frail, vulnerable and had complex health needs (38). As reported in this study, much of the resources used in caring for them devoted to the management of their chronic illnesses and acute exacerbation of their conditions (39, 40). Before the implementation of SMOP, the nursing homes looked after their residents' medical condition often “in silo”. For residents who had multiple comorbidities attended by various specialists, there needed to be frequent transportation arrangement to take and accompany them to the hospitals for specialist consultations and prescription medicines. The nursing homes also faced great challenges in providing appropriate healthcare for their residents with complicated conditions to reduce hospitalization and emergency room visits (38, 41).

By implementing the SMOP, some of the immediate benefits experienced by the nursing home staff included the partnerships with specialists from the hospitals in coordinating the care for residents with complex health conditions. Similar to the Hospital in the Nursing Home model of care in Australia (42) where out-of-hospital healthcare services was provided for older people, SMOP specialists paid visits to the nursing homes on a regular basis to work with the frontline healthcare staff. An essential element of such partnership was the exchange of patient information and inter-disciplinary communication across the care settings (43). Participants in this study repeated their long-standing concerns about lacking access to the information about the residents' diagnosis, test results and treatment recommendation made in the hospitals, offering challenges to their care-giving process.

Indeed, poor communication between nursing home and hospitals has been cited as the main cause of impeding the continuity of care. Improved information exchange with hospital services in the form of direct communications, similar to what had been previously reported (44), had been shown to be positively associated with staff satisfaction when delivering care for the residents. Standardized information transfer forms (45) and effective utilization of information and communication technology (46, 47) should be further encouraged for fostering the two-way communication across different care settings For instance in Japan, the Mame-net developed by the local government to share acute and chronic changes in the residents' medical conditions had been shown to improve nursing home care and reduce emergency transportation to hospitals (48).

Having access to geriatric and other specialists' expertise through SMOP was also found beneficial to the decision making about care for the residents at the nursing homes in this study. Many participants mentioned that they often encountered challenging situations when caring for residents with complex health issues, a common concern for many healthcare staff at the nursing home settings (49). Appreciation was shown by the participants to the SMOP team who shared their expertise knowledge during the visits through case discussions and other formal encounters. Direct contact also helped building and extending the professional network enabling more seamless professional exchanges even in informal settings. Similar to previous findings (50), the professional exchange fostered by partnership-based collaboration was helpful to the healthcare staff at the nursing homes to handle and resolve difficult resident scenarios and to acquire new strategies to deal with challenging situations.

Transfer of knowledge between the SMOP team and the nursing home staff was also considered an opportunity of capacity building by many participants. As previously reported, similar outreach interventions which featured coordination of care and access to skilled healthcare providers were often associated with capacity building demonstrating some positive effects on the quality improvement of the nursing homes (51, 52). Nursing home healthcare workforce being equipped with good clinical knowledge and communication skills, competent and engaging is critical to realizing the long-term outcome of the SMOP and other outreach programs alike (53, 54). As improved teamwork and communication has been proven to enhance the quality of care in the nursing homes (55, 56), dedication of more time in teamwork training for nursing home staff through SMOP was deemed important.

For practical reasons, the nursing home participants emphasized on the benefits of SMOP in reducing the burden of transportation arrangement needed for mobilizing residents since regular returns to their specialists' offices for follow-up consultations were no longer needed for residents under the care of the SMOP team. This was considered a significant advantage in terms of reducing the workload for the frontline staff and rationalizing the use of highly limited resources for the nursing homes. More importantly, the SMOP team composed of multi-disciplinary specialists who attended to the same resident at the same time during their visits, and the frontline staff at the nursing home were able to communicate with the specialists directly which would be not possible otherwise. Similar to related programs (57), this encouraged cross-setting collaboration which could be beneficial in achieving more comprehensive monitoring of the residents' health conditions and promoting teamwork for a patient-centered care approach in the long run.

Limited by the nature of this study, reported positive effects of SMOP on the clinical outcomes for the nursing home residents (e.g., reduced adverse events and improved disease control) were only speculations by the participants. There were already requests from the nursing homes for more frequent visits by a larger inter-disciplinary SMOP team. Indeed, as previous studies reported, outreach programs could easily achieve a high level of satisfaction and convenience by the care recipients (58, 59). However, very little discussion was directed to the need and know-how of assessing the SMOP outcome, reflecting a common deficit in recognizing the importance of monitoring and evaluation for the sustainable development and continuous improvement of the health program (60). It is yet to determine if outreach programs like SMOP could become powerful mechanisms for addressing complex public health problems by leveraging the resources and expertise from the public sector (61).

The ultimate goals of the SMOP model were to improve the residents' health outcome and thus reduce the need for acute care in the emergency department and hospital setting. According to the government statistics, a reduction in emergency department visits by 10.5% and emergency admission by 15.5% had been observed after the implementation of SMOP in 2020 in comparison to the previous year (62). However, for quality improvement and sustainable development purposes, high quality evidence from well-designed studies that collect both quantitative and qualitative data are needed to establish a robust assessment framework. Indicators such as decrease in medical admissions, hospital admissions, mortality ER admissions, adverse drug events length of hospital stay could be used to measure the effectiveness of the SMOP (43, 63).

At the same time, the importance of cost-effectiveness of SMOP should be prioritized especially considering the continuous economic challenges pertained to the COVID-19 pandemic. Involving multiple specialists in the SMOP and programs alike is a high-cost exercise (58, 64). The cost-effectiveness analysis of the SMOP should also be considered as one of the key measures to determine if the SMOP was cost-saving and to inform decisions on allocation of healthcare resources for the future development of the SMOP (26, 65).

## Strengths and limitations

The interviews were conducted with a range of key stakeholders from eight out of the 11 nursing homes participating in the SMOP in Macao. The insights unfolded represented a rich, representative perspective about how such program was implemented and the outcome interpreted. All the interviews were conducted face-to-face by an experienced research team in a standardized manner.

On the other hand, this study also had a number of limitations. Firstly, this study only involved the key stakeholders from the service-receiving end so the findings could only inform the current perspectives partly. Secondly, there could be risks of social desirability bias based on the following consideration: (1) due to the nature of the SMOP, which was operated by the government and freely provided to the needy residents in the nursing home, participants might have invested interests and their opinions might skew toward positive comments of possibly resulting in incomplete sharing of negative feedback; and (2) due to the nature of qualitative research, the researchers' presence during interviews might affect the way participants responded to the questions. Thirdly, 3 of the 11 nursing homes participating in the SMOP did not participate in this study. These non-participating nursing homes were relatively smaller in scale in terms of resident number and nursing staff. As such, the experiences of SMOP implementation and outcome might differ.

## Conclusions

Interviews revealed multiple benefits of the SMOP initiative in driving patient-centered care from the perspective of the participating nursing homes. The areas of improvement identified were multifaceted and strongly indicate there is a need for a concerted and collaborative effort between members of the SMOP team and the staff of the nursing homes. For this, efficient data-sharing and communication mechanisms are crucial to improve the continuity of care across the sectors. Future actions are needed to yield the insight from the service-providing perspective (i.e., the SMOP team members and the decision-makers). In addition, further studies are warranted to further explain what in the SMOP works best, for whom and under what circumstances, and how to make SMOP more effective. In order to inform resources allocation for the continuous development of SMOP, parameters tailored to the nursing home residents should be defined for informing a robust assessment framework to demonstrate the cost-effectiveness of the program.

## Data availability statement

The original contributions presented in the study are included in the article/Supplementary material, further inquiries can be directed to the corresponding author/s.

## Ethics statement

The studies involving human participants were reviewed and approved by the Research Ethics panel of the University of Macau (reference number: SSHRE21-APP001-ICMS). The patients/participants provided their written informed consent to participate in this study. Written informed consent was obtained from the individual (s) for the publication of any potentially identifiable images or data included in this article.

## Author contributions

ZC and JL carried out literature review, collected and handled data, prepared data for analysis, interpreted the results, and drafted the manuscript. HH designed the study, supported data analysis, and critically reviewed the manuscript. KL, CL, ZL, and TC collected and handled data, prepared data for analysis, and supported the writing of the manuscript. CU conceptualized the research, designed the study, verified the data analysis results, and critically reviewed the manuscript. All authors approved the final manuscript.

## Funding

This study was funded by the University of Macau (SRG2021-00007-ICMS) and the Science and Technology Development Fund, Macau SAR (0006/2020/AKP and SKL-QRCM (UM)-2020-2022).

## Conflict of interest

The authors declare that the research was conducted in the absence of any commercial or financial relationships that could be construed as a potential conflict of interest.

## Publisher's note

All claims expressed in this article are solely those of the authors and do not necessarily represent those of their affiliated organizations, or those of the publisher, the editors and the reviewers. Any product that may be evaluated in this article, or claim that may be made by its manufacturer, is not guaranteed or endorsed by the publisher.
