# Supplementary material for: Exploring the implementation of an outreach specialist program for nursing home residents in Macao: A multisite, qualitative study
Source: Front Public Health. 2022 Sep 29;10:950704. doi: 10.3389/fpubh.2022.950704 (PMC9558699; doi:10.3389/fpubh.2022.950704)
Supplement: Supplementary file 1 [file Data_Sheet_1.docx]

**Supplementary file 1**

**Exploring the implementation of an outreach specialist program for nursing home residents in Macao: a multisite, qualitative study**

**Interview Protocol**

Each interview was conducted following an Interview Protocol designed for this study:

1. Prior to the start of each interview, the investigators shared a copy of the Participant Information Statement with the participant and explained to him/her again the background and the objective of this study.
2. The participant was asked to provide a signed informed consent to their participation and the audio-recording of the interviews.
3. At commencement, the leading investigator introduced the interview team and their roles during the interview.
4. Participants were asked to provide their background information including their role in the nursing home, and the number of years working in the nursing home.
5. During each interview, 1 investigator led the conversation with the participant while 2 other investigators were responsible for recording field notes, key discussion points and non-verbal expression of the participant.
6. The semi-structured interview covered at least the following questions:
   1. Could you please share your experience of the SMOP program in your facility?
   2. What would you consider the most critical impact of the outreach program on your facility?
   3. What are the most noticeable outcome of the outreach program to people in your role and the residents you looking after?
   4. What are the areas of concerns about the outreach program?
   5. How would you see the outreach program develop in the future, and what could be the most important factors?
7. The interviews lasted for 20-50 minutes (an average of 35 min) and were audio-recorded and conducted until saturation was reached for the key emergent themes.
