# Supplementary material for: Exploring the implementation of an outreach specialist program for nursing home residents in Macao: A multisite, qualitative study
Source: Front Public Health. 2022 Sep 29;10:950704. doi: 10.3389/fpubh.2022.950704 (PMC9558699; doi:10.3389/fpubh.2022.950704)
Supplement: Supplementary file 2 [file Data_Sheet_2.docx]

**Supplementary file 2**

**Exploring the implementation of an outreach specialist program for nursing home residents in Macao: a multisite, qualitative study**

**Data analysis Protocol**

The analysis of data (including the transcript, observation notes and the audio-recordings) was conducted as follows:

1. Z Cen and J Li separately listened to all the interviews and read the transcripts and observation notes multiple times, recorded key ideas and recurrent themes as they emerged, and generated initial codes to capture the meaningful fundamental element of the data.
2. Z Cen and J Li developed a coding system (which provided a brief definition of each code) together which was then confirmed by H Hu and COL Ung. A Microsoft Excel file was developed for the codes of the transcripts to be tabulated and organized.
3. Each transcript was coded line by line by 2 of the 6 investigators (Z Cen, J Li, KC Lei, CI Loi, Z Liang, TF Chan) separately. The coding results of each transcript was recorded using the Excel file, compared and negotiated among the pair. Either H Hu or COL Ung was consulted in case of any disagreement in the coding results.
4. The coding results of all the transcripts were pooled, and all the codes were categorized into themes and sub-themes by the 6 investigators (Z Cen, J Li, KC Lei, CI Loi, Z Liang, TF Chan) together.
5. H Hu and COL Ung separately assessed the integrity of the process of data collection, analysis, interpretation and theme generation and the potential impact of reflexivity.
6. To ensure credibility, dependability and confirmability of the findings, member check of the coding results of individual transcripts and the pooled dataset was offered to the participants. None of the participants said they had the time to check the transcript so no transcript was returned to participants for review.
