# Supplementary material for: Exploring the implementation of an outreach specialist program for nursing home residents in Macao: A multisite, qualitative study
Source: Front Public Health. 2022 Sep 29;10:950704. doi: 10.3389/fpubh.2022.950704 (PMC9558699; doi:10.3389/fpubh.2022.950704)
Supplement: Supplementary file 3 [file Data_Sheet_3.docx]

**Supplementary File 3.**

Thematic analysis of the qualitative data with supporting quotes

| **Themes, subthemes and codes** | **Supporting quote 1** | **Supporting quote 2** |
| --- | --- | --- |
| **Theme 1: Overall perception about the SMOP** | | |
| Subtheme 1.1:  Addressing the needs  *Codes:*   1. *Lack of expertise* 2. *Lack of manpower* 3. *Time constraints* 4. *Difficulties in logistics arrangement* 5. *Transportation challenges* 6. *Waiting time* | *“The manpower and transportation is one thing. You see, most of our residents cannot move around easily or even have troubles sitting. And there is usually a long waiting time at the specialist office. We are so pleased to have the SMOP now and to see how the SMOP has helped us and our residents where we need the help the most.” (Nursing home 5_07, manager)* | *“The SMOP sent specialists including geriatrics, psychiatry, orthopedics, and surgeons from the public hospital. The integrated expertise support is exactly what we need to care for our very sick residents.” (Nursing home 4_01, manager)* |
| Subtheme 1.2:  Sharing information among caregivers  *Codes:*   1. *Blood test results* 2. *New diagnosis* 3. *Changes in treatment* 4. *Adverse drug reactions* 5. *New signs and symptoms* 6. *New health concerns for the residents* | *“Before when we did not even have the access to our residents’ latest blood test results in the hospital, not to mention the opportunity to get hold of the specialists. Now we can actually discuss about the cases and decide on the treatment together whenever the SMOP team is here.” (Nursing home 5_01, doctor)* | *“The opportunity is for us to share with the pharmacists in the SMOP team any adverse drug events we observe especially when new medicines are prescribed by the specialists. Having a pharmacist in the SMOP team means that we can now work closer together to ensure drug safety.”(Nursing home 7_16, pharmacist)* |
| Subtheme 1.3:  Access to the experts  *Codes:*   1. *Visits by specialists* 2. *Access to specialists’ advice* 3. *Cross-sector collaboration* 4. *Learning opportunity* | *“Outreach specialists include geriatrics, psychiatry, internal medicine, surgeons, etc. Geriatric specialists come here most often, about twice a month, and psychiatric specialists come once a month. They visit almost ten to twenty residents each time. Discussion with different specialists at the same time is also a very good leaning opportunity for us.” (Nursing home 1_01, doctor)* | *“Sometimes the clinical pharmacist in the SMOP team provide advice about the management of our pharmacy room. Sometimes we can check with them about which medicines can be crushed. Pharmacy staff can also learn from them how to evaluate and optimize the drug regimens.” (Nursing home 5_02, pharmacy technician)* |
| **Theme 2: Benefits of the SMOP as experienced or expected** | | |
| Subtheme 2.1:  At the nursing home level  *Codes:*   1. *Quality of care* 2. *Safety* 3. *Effective use of resources* 4. *Accuracy* 5. *Patient-centered care* 6. *Effective use of resources* | *“The SMOP team includes the psychiatry, geriatrics, general internal medicine and urology specialists. The specialist care came to us so that our residents do not need to visit the specialists one by one. Of course it will help improve the medical care for our needy residents. More importantly, the SMOP saved us a lot of resources and manpower to transport our residents to the hospital and back. Such resources can be diverted to cover other areas of concerns” (Nursing home 6_06, nurse assistant)* | *"Prior to the visit by the SMOP team, there is always some preparation work such as summarizing the clinical and nursing notes, and checking for any potential issues. Of course the SMOP team will do the assessments again. If there are any issues, the SMOP specialists will discuss with our residents doctors. After the SMOP team visit, we will need to update the residents’ files and treatment plan as needed. So you see, the SMOP has actually prompted us to pay closer attention to the most needy residents before, during and after the SMOP team visit. In a way, the SMOP helps us double or even triple check our services." (Nursing home 1_04, manager)* |
| Subtheme 2.2:  At the resident level  *Codes:*   1. *Early detection of critical health issues and timely referral* 2. *Reduced unplanned hospital admission* 3. *Improved disease control* 4. *Improved clinical outcome* 5. *Reduced risks of adverse events* 6. *Reduced visits to ER* 7. *Reduced hospitalization* | *"The decision is based on whether the residents are seriously ill or bedridden for a long time. In addition to the planned residents to be visited, the team also will visit other residents who indeed need a visit. If there were an urgent situation, we would discuss with the SMOP team to try to resolve the problem sooner." (Nursing home 7_03, manager)* | *"If there were any problems, the nurses will contact the SMOP specialists on the phone and find out what to do. Sometimes outreach doctors make timely judgments and suggest that we send high-risk residents to the hospital-based on the situation. But more often, potential issues can be picked up much sooner through discussion for actions to be taken in a timely manner to avoid deterioration of the health conditions and the residents did not have to end up in the emergency room."( Nursing home 6_05, nurse assistant)* |
| Subtheme 2.3:  At the professional level  *Codes:*   1. *Communication* 2. *Collaboration* 3. *Inter-disciplinary healthcare team* 4. *Capacity building for nursing home staff* 5. *Timely update of residents’ conditions* 6. *Reduced error in transferring residents’ clinical data* | *“Before the SMOP, there was no way to find out the residents’ latest blood test results or other clinical measurements. Of course we could always make a call but it was very inconvenient and did not always work. The SMOP is an opportunity for us to get access to the residents’ latest clinical readings especially after recent discharge so that our decision about changing medicines or the dosages can be more informed and we can look after our residents more appropriately.” (Nursing home 2_02, nurse)* | *“We know about the residents’ daily life better than anyone even their families. During the SMOP visits, I can directly talk to the doctor about residents' situation. We are able to communicate with the specialist about the residents’ conditions and what medications to use or what treatments to use. I believe our input is also useful for the specialists’ decision-making. It feels like we have a partner to communicate and work with.” (Nursing home 8_01, doctor)* |
| Subtheme 2.4:  At the health system level  *Codes:*   1. *Integration of healthcare resources* 2. *Prioritization of healthcare resources* 3. *Improvements in elderly care* 4. *Reduction of avoidable burden on healthcare system* 5. *Prevention of drug wastage* 6. *Capacity building for nursing home staff and SMOP team members* | *“The SMOP formed the specialist team based on the needs of the high-risk residents. At present, geriatrics and psychiatric specialists are the main ones. I understand that orthopedic specialist will also join the SMOP team. Every time they visit, they will prioritize their time to the residents who have been previously screened as high risk. Whenever they have time, they will also check on residents who are more seriously ill, or bed-ridden. In a way, they also take the chance to help reduce the use of health resources in the hospital setting. I can see that the SMOP continues to evolve to better match our needs.” (Nursing home 5_01, doctor)* | *"When the outreach team comes, we will talk to the outreach doctors about the patient's discomfort and the need to adjust the medicine taking. We will tell him the necessary changes to the drug regimens. Working with the SMOP team means that we are at a better position to help optimize the treatment regimen, ensure the proper use of medicine and minimize any drug wastage for our residents." (Nursing home 4_01, manager)* |
| **Theme 3: Areas of improvement of SMOP** | | |
| Subtheme 3.1:  The design of the SMOP  *Codes:*   1. *Frequency of visits* 2. *Involvement of more specialists in the SMOP team* 3. *Greater support for psychiatric and mental health of the residents* 4. *Case discussion* | *"I think we need more support especially for residents with mental health disorders. We have quite a few of them here and their condition may change at any time. The SMOP team only come to visit every 2 or 3 months and sometimes the residents’ conditions become more serious between the visits. I hope the SMOP team can visit us more often and we can get more support for residents with mental illnesses.” (Nursing home 3_03, nurse)* | *"The SMOP team has already helped us a lot because different specialists have already been involved to cover most of the areas. At the moment, the support for the residents with psychiatric disorder is not enough. What I think is needed is to have psychiatric specialists to visit our residents more frequently. Of course, to the residents, having more specialists in the SMOP team is of course better." (Nursing home 8_03, pharmacy technician)* |
| Subtheme 3.2:  Preparation of the nursing home  *Codes:*   1. *Better workflow* 2. *Staffing arrangement prior to and during SMOP team visits* 3. *Preparation of residents’ health records* 4. *Screening criteria for residents receiving SMOP services* | *“Usually the SMOP team would screen for the high-risk residents and notified us the list of residents. Once we are notified, we need to prepare the residents’ files including the recent clinical notes and medication lists. The preparation work of the SMOP team visit is a time-consuming task and adds on the work pressure that we already have to endure. Better workflow or even IT tools are needed to help alleviate our workload.” (Nursing home 8_02, nurse)* | *"Every time when the SMOP team visits, I will try to make myself available to join in the discussion. I personally would like to communicate with the SMOP pharmacist more frequently, but I might not be able to spare the time due to other work arrangement. I am trying to adjust the staffing so that our frontline staff are able to work with and benefit from the SMOP team whenever they visit." (Nursing home 4_01, manager)* |
| Subtheme 3.3:  A continuous communication and data sharing mechanism  *Codes:*   1. *Feedback loop* 2. *Cross sector communication* 3. *Data sharing mechanisms* 4. *Access to electronic medical record* 5. *Means of cross-sector communications* | *"At present, we only can get in contact with the residents' specialists through the SMOP. The SMOP team can help convey the key messages about the residents’ conditions to their specialists in the hospital. The specialists from the hospital might relay an update to us through the SMOP team. The information conveyed is very limited and simple, the whole process is relatively indirect, and it is easy to make mistakes. I think, with the residents’ consent, having a proper data sharing mechanism is important so that we can share, update and get access to the residents’ key clinical notes and blood test results." (Nursing home 2_02, nurse)* | *“I can see that effective communication between our doctor and other healthcare providers with the SMOP team is really valuable for the continuous care for the residents especially those who often need hospital care and surgical procedures. Given the long waiting list to specialty consultation or surgery, if there is already a communication mechanism so that alert can be raised in case of raised urgency, the management of the residents can be much better informed and, whenever appropriate, prioritized.(Nursing home 5_08, manager)* |
